# Biomass-Derived Production of Itaconic Acid as a Building Block in Specialty Polymers

**DOI:** 10.3390/polym11061035

**Published:** 2019-06-11

**Authors:** Bernadette-Emőke Teleky, Dan Cristian Vodnar

**Affiliations:** 1 Institute of Life Sciences, University of Agricultural Sciences and Veterinary Medicine, Calea Mănăştur 3-5, 400372 Cluj-Napoca, Romania; bernadette.teleky@usamvcluj.ro; 2 Faculty of Food Science and Technology, Institute of Life Sciences, University of Agricultural Sciences and Veterinary Medicine of Cluj-Napoca, Calea Mănăștur 3-5, 400372 Cluj-Napoca, Romania

**Keywords:** itaconic acid, biotechnology, biosynthetic pathways, *Aspergillus terreus*, polymers, hydrogels, drug delivery

## Abstract

Biomass, the only source of renewable organic carbon on Earth, offers an efficient substrate for bio-based organic acid production as an alternative to the leading petrochemical industry based on non-renewable resources. Itaconic acid (IA) is one of the most important organic acids that can be obtained from lignocellulose biomass. IA, a 5-C dicarboxylic acid, is a promising platform chemical with extensive applications; therefore, it is included in the top 12 building block chemicals by the US Department of Energy. Biotechnologically, IA production can take place through fermentation with fungi like *Aspergillus terreus* and *Ustilago maydis* strains or with metabolically engineered bacteria like *Escherichia coli* and *Corynebacterium glutamicum*. Bio-based IA represents a feasible substitute for petrochemically produced acrylic acid, paints, varnishes, biodegradable polymers, and other different organic compounds. IA and its derivatives, due to their trifunctional structure, support the synthesis of a wide range of innovative polymers through crosslinking, with applications in special hydrogels for water decontamination, targeted drug delivery (especially in cancer treatment), smart nanohydrogels in food applications, coatings, and elastomers. The present review summarizes the latest research regarding major IA production pathways, metabolic engineering procedures, and the synthesis and applications of novel polymeric materials.

## 1. Introduction

The use of non-renewable petrochemicals still leads today`s petrochemicals industry, while biomass is the only renewable source of organic carbon on Earth. Organic acid production through microbial fermentation of different biomass wastes can play an essential role in the production of biochemical building-blocks or even bioactive compounds [1,2,3,4]. Isikgor & Becer [5] have recently presented over 200 significant compounds derived from different biomass sources and structures with pretreatment methods that can also reduce the production costs of chemicals and polymers.

Lignocellulosic biomass such as energy crops, agricultural and forest management residues, and municipal wastes are versatile renewable energy sources [6]. They can potentially replace fossil fuels in power and heat generation, and natural gases in the production of bio-based chemicals. A recent review analyzed the present situation of bio-based chemical production through biological and chemical pathways, presenting 435 chemicals and materials obtained from renewable resources [7]. Biomass and biomass-derived wastes have the potential to provide low-cost sources of sugar and could be the best substitute for non-renewable petrochemicals.

A current major problem is the high amount of plastics present in the environment and their role in environmental pollution, since they don’t degrade under natural circumstances. These plastics are mostly composed of synthetic polymers derived mainly from petrochemicals. A solution for this never-ending problem is the exploration of alternative bio-based and biodegradable plastics like biosynthetic polymers [8], polylactic acid (PLA), thermoplastic starch (TPS) or even natural polyesters like polyhydroxyalkanoates (PHA) [9,10].

One of the most important classes of compounds obtained from lignocellulose biomass are organic acids. Bio-based organic acids are products that are derived from different biomass sources, which are sustainable, cost-effective, and environmentally friendly. Among these, itaconic acid (IA), together with its derivatives, is an essential renewable chemical because it has various uses in the pharmaceutical and food industry, and also presents a feasible substitute for unsaturated acids like acrylic, methacrylic, maleic, fumaric acid and their derivatives [11,12,13]. Integration of IA in polymers is very efficient [14].

**Figure 1:**
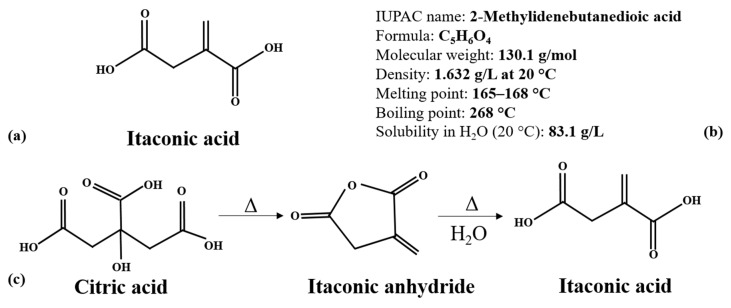
The chemical structure (**a**), properties (**b**) and chemical synthesis from citric acid (**c**) Δ: heat input.

IA is an unsaturated dicarboxylic acid (C_5_H_6_O_4_), also known as 2-methylenebutanedioic acid, propylene dicarboxylic acid, or 2-methylenesuccinic acid (Figure 1a,b). IA is highly soluble in water and alcohols [15], stable at average temperatures, and, being a weak acid, it is also stable in middle-basic, neutral and acidic conditions [16]. It has an appearance of white crystalline powder or crystals, and it is odor-free [17,18]. The variation of IA’s functional groups makes it an efficient intermediate to produce different complex organic compounds. It can participate in a wide variety of reactions like esterification with alcohols, salt formation with metals, production of anhydride, polymerization, and additional reactions [19].

Baup S. discovered IA in 1837 as a product of thermal decomposition of citric acid [20], while Kinoshita was the first to report production of IA with *Aspergillus itaconicus* in 1931 [21]. Later, the focus for IA fermentation was shifted mostly to *A. terreus* strains. Nelson et al. [22] studied *A. terreus* NRRL strain 1960 and established a biotechnical process; while Eimhjellen et al. [19] studied the effect of different substrates (various sugars and alcohols) on IA production. Production with *A. terreus* NRRL 1960 in 20 mL media and 5% substrate in 100 mL flasks resulted in the highest IA production with sucrose (57%) and d-glucose (52%). Other substrates like cellobiose (41%), d-mannose (32%), d-xylose (31%), d-fructose (26%) and glycerol (23%) produced significant amounts of IA as well [23].

The initial industrial production of IA used a chemical approach, i.e., the pyrolysis of citric acid to itaconic anhydride, followed by the hydrolysis of the anhydride (Figure 1c) [20]. Alternative methods were decarboxylation of aconitic acid, dry distillation of anhydride, and oxidation of isoprene [24,25]. Due to the numerous steps and low production rates, the chemical synthesis of IA is not very effective in comparison with the biotechnological method [26].

Numerous reviews have summarized the attempts to improve the various IA production technologies [27,28]. Many researchers have actively evaluated different industrially significant microorganisms that synthesize IA [29,30], and also specific improvements through genetic engineering of various strains for higher IA production [31,32]. Due to the increased global marketing potential of IA, biotechnological production was also evaluated [33]. Different polyesters, bio-based resins, and other commodity materials were also highly considered [14,34], showing the increasing demand for IA yield improvement and for lower production costs. According to the US Department of Energy, IA is included in the top 12 building block chemicals, since it is a promising platform molecule with extensive applications [35,36].

The production of IA (around 41,000 tons/year) takes place currently mainly in three countries: the USA, China, and India [11,36,37,38]. The high request for bio-based materials anticipates a further growth of 60% of the IA world market, predicted to surpass 216 million USD in 2020 [33,39]. Assuming that IA can replace polyacrylic acid, the market value would increase even more, and IA would become a valuable compound in the industrial biotechnology field [30,40].

To compete with conventional chemical methods, bio-based processes yielding chemical building-blocks should have a production rate of 50 g/L, 80% theoretical yield and a volumetric capacity of about 3 g/L [35,41]. Biorefinery improvement has two key roles: an economic goal by decreasing the production price, and a strategic goal to supplement the petroleum-based industry by exploiting the renewable biomass residues [41].

The aim of this review is to summarize recent advancements in biotechnological IA production in terms of substrates and optimal conditions used, the progress made in metabolic engineering and the state-of-the-art in major IA applications.

## 2. IA Synthesis by Microbial Fermentation

The most commonly used fungi for IA production are the *Aspergillus* species [42,43], and mainly *A. terreus* [37,44,45]; other microorganisms are also able to produce IA, e.g., the filamentous fungus *Ustilago maydis* [29,46,47] and even species of *Pseudozyma*, although only *P. antarctica* was shown to produce a small quantity of IA. Using *P. antarctica* Y-7808 under nitrogen-limited conditions, the IA production on different carbon sources was 11 g/L for fructose, 10 g/L for glucose, 8–9 g/L for sucrose and 6–7 g/L for maltose [48].

### 2.1. Biosynthetic Pathways

**Figure 2:**
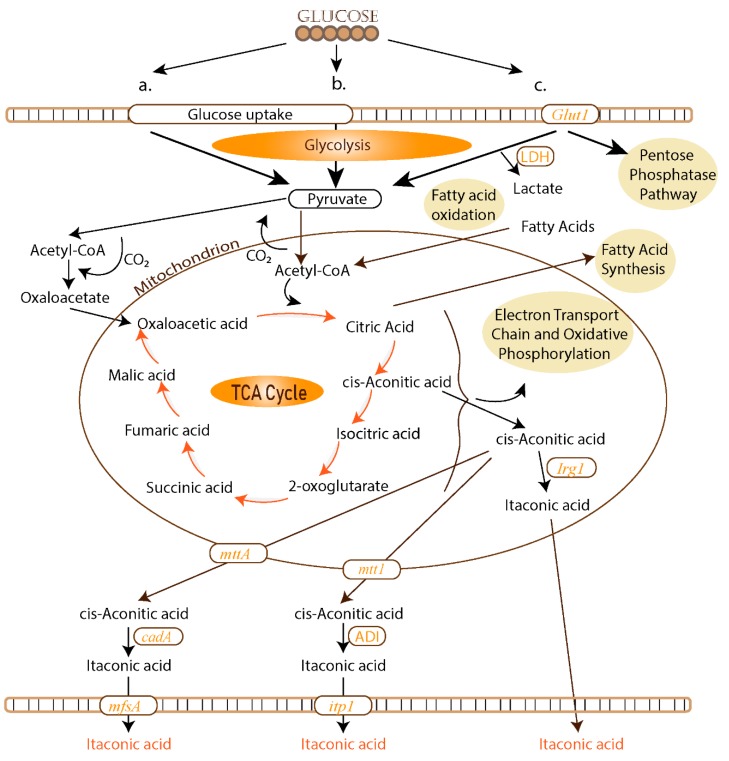
Biological pathway of IA in (**a**) *A. terreus*, (**b**) *U. maydis*; and (**c**) in macrophages.

The IA biosynthesis in *A. terreus* takes place under aerobic conditions and begins with glucose, which is transformed to pyruvate via glycolysis (Figure 2a). Pyruvate is metabolized to acetyl-CoA, and a carbon dioxide molecule is released. A partial transformation of acetyl-CoA to oxaloacetate occurs with the inclusion of the formerly liberated carbon dioxide molecule prior to metabolizing acetyl-CoA in the mitochondrion. From oxaloacetate and acetyl-CoA, citrate and cis-aconitate are produced in the citric acid, also known as tricarboxylic acid (TCA) cycle. Next, the mitochondrial tricarboxylate transporter (*mttA* gene) transfers cis-aconitate in the cytosol. There, cis-aconitate decarboxylase (encoded by the *cadA* gene) facilitates the production of IA by discharging carbon dioxide [49,50,51]. Simultaneously, the facilitator superfamily protein (*mfsA* gene) transfers IA outside the cell. Therefore, the genes that contribute to the IA production are *cadA*, *mttA*, and *mfsA.*

*U. maydis* presents an alternative biosynthetic pathway for IA production (Figure 2b) that shows a different course mainly because it does not express the *cadA* gene. The mitochondrial tricarboxylate transporter (*mtt1* gene) secretes cis-aconitate to the cytosol, where it is decarboxylated to itaconate by the cytosolic aconitate-Δ-isomerase (ADI). Itaconate is afterwards excreted from the cytosol to the external medium through the cell wall by the membrane transport protein ITP1 (major facilitator) [47].

IA, defined as an essential anti-microbial agent, is also produced in mammalian macrophages (Figure 2c) using the immunoresponsive gene 1 (*Irg1*) which codes for the enzyme (cis-aconitate decarboxylase) responsible for IA production [52,53]. The production of IA in macrophages takes place via a TCA intermediate during decarboxylation of cis-aconitate [54,55].

### 2.2. IA Fermentation Using Different Substrates and A. terreus Strains

The principal producer of IA is *A. terreus.* So far, many research studies have been published on IA fermentation with different strains of this fungus (Table 1). A wide variety of *Aspergillus* species are used in the production of many primary and secondary metabolites (e.g., lovastatin, terrein, cyclosporine, and asperfuranone). *A. terreus* is a saprotrophic fungus inhabiting soil and has both damaging (adverse health effects in plant crops and humans) and favorable effects (from a biotechnological perspective) [56].

One of the limiting aspects of IA production is the high cost of the carbon source [37]. Glucose is the most frequently used substrate, however, not the cheapest carbon source [36].

Due to the high amount of different lignocellulose-rich waste materials providing an energy potential of around 12–120 EJ (exajoules) [57,58,59], these possible sources of raw materials have recently received special attention. Promising substrates are various lignocellulose hydrolysates (i.e., wheat bran, beech wood, rice husk), starch and molasses, which could be a good alternative source of glucose. A recent case study of itaconate synthesis from pretreated and hydrolyzed beech wood established a reasonable production yield, practicability, operation mode, and the favored microorganism. The final yield of obtained IA was with *A. terreus* 0.35 g IA/g glucose, and with the genetically engineered *U. maydis* 0.48 g IA/g glucose. In this study, the genetically enhanced *U. maydis* was the preferable microorganism for IA production from pretreated biomass due to medium contamination tolerance. On the grounds of advantages and drawbacks of biomass transformation to itaconate, future improvements were also highlighted [60].

Instead of glucose, the use of starch-rich wastes (corn, rice, and potato starch) as substrates with different fungi provide enhanced IA production [61]. In a recent study, the enzymatically hydrolyzed substrates resulted in 180 g/L glucose. With *A. terreus* strain, the highest IA production obtained was C1 (30.8 g/L) and C2 (23.4 g/L) with potato as waste [36]. Cornstarch is another very pure, economical, and balanced carbon source in mass production. Because it is an outstanding substrate, it was also used for fermentation with *A. terreus* TN-484 in a hydrolyzed form, with >60 g/L IA yield. By overexpressing the glucoamylase gene in a genetically engineered *A. terreus*, the production rate increased to 77.6 g/L [62].

A possible substrate for IA production with two strains of *A. terreus* (ATCC 7860 and ATCC 10020) is pretreated rice husk hydrolysate [63]. After rice husk pretreatment, the concentration of monosaccharides: glucose, xylose, and arabinose decreased from 35.66%, 14.79% and 3.61% to 17.18%, 0% and 0%, respectively. Using different concentrations of these monosaccharides, six different media were prepared and subjected to fermentation for the optimization of IA production. The results indicated that rice husk can be used through acidic phosphoric hydrolysis with *A. terreus* and produce 1.9 g/L IA from 35 g/L glucose. These results indicate that the favored source of carbon in IA production is glucose. In a wheat bran hydrolysate, *A. terreus* CICC 40,205 was used in optimized conditions (pH 7, 32 °C) and a final concentration of 49.65 g/L IA was obtained [64].

By using second generation feedstock (wheat chaff) and alkaline pretreatment (NaOH) following enzymatic saccharification (carbohydrate polymer transformation in free monomeric sugars) with *A. terreus* DSM 23,081, the production of IA was 27.7 g/L [45,65]. Using the same filamentous fungus strain, but glucose as substrate, the outcome was 86.2 g/L itaconate [66]. Saha and Kennedy analyzed six distinctive *A. terreus* strains and also different sugars as substrates (glucose, xylose, arabinose, and mixed sugars) [67]. The highest IA production was 47.9 g/L using glucose as substrate and *A. terreus* NRRL 1961 strain; from xylose, the highest quantity was 36.0 g/L IA with *A. terreus* DSM 23,081 strain.

Hevekerl et al. studied the effect of pH on IA yield [68], and the highest concentration of itaconate achieved was 146 g/L at pH 3 with *A. terreus* DSM 23,081 strain. The initial pH has to be low for the microorganisms to achieve IA production capability [19]. Using optimized conditions, the concentration obtained by Larsen and Eimhjellen [23], was 129 g/L, which is a very high concentration compared to the results of other investigations (Table 1).

As observed in various studies, different parameters influence the final production of IA: the used microorganisms, temperature, medium concentrations, the initial pH and the variation of pH values through the fermentation process, oxygen supply and also the formation of side products [68,69]. Therefore, the creation of competent microbial cell factories for IA production through genetic engineering should bring substantial benefits for the production levels [62,70].

### 2.3. IA Fermentation with Other Microorganisms

The primary production of IA is currently done with *A. terreus* strains, but other microorganisms like Ustilago sp. [47,79], Candida sp. [80] and Pseudozyma sp. also produce IA [48] (Table 2).

Although *U. maydis* produces lower amounts of itaconate (maximum reported at 34.52 mg/L) compared to *A. terreus*, this fungus integrates essential assets of yeast and filamentous fungi, like the growth in a yeast-related morphology in haploid form of single cells. *U. maydis* MB 215 is an encouraging IA producer on pretreated lignocellulosic wastes (beech-wood). The production of IA was achieved predominantly on three differently pretreated substrates (enzymatically hydrolyzed cellulose, salt-assisted organic acid catalyzed cellulose and hydrolyzed hemicellulose), although genetic alteration could have the capacity to increase the produced quantity [36].

Tabuchi et al. [80] could not identify the used *Candida* yeast strain, but with pH values above 3, the preliminary results showed that IA production was within the range of 30–35 mg/mL.

IA production with *P. antarctica* NRRL Y-7808 in nitrogen-restricted growth conditions in culture flasks was 30 g/L, starting from 80 g/L glucose. Other *Pseudozyma* strains did not produce IA, so this is not a common feature of this yeast [48].

The global development of biodiesel production results in high raw glycerol content, a primary byproduct of biodiesel manufacturing [89]. Through glycerol fermentation, numerous valuable compounds can be obtained [90,91,92]. Zambanini et al. [82] studied the production of IA from glycerol with the *U. vetiveriae* strain TZ1 and also with the same strain but through overexpression of *ria1* and *mtt1* genes. Quantities achieved were above 34.7 g/L itaconate from 196 g/L glycerol using a medium optimization in shake flasks buffered with CaCO_3_.

### 2.4. Metabolic Engineering

Metabolic engineering has unlocked an innovative path and is used to improve the genetic and regulatory mechanisms inside cells to expand IA production [49,62,93,94].

Through genetically engineered *A. terreus* strains, there is a possibility to achieve a considerable shortening of the fermentation process during the integration of the two phases, saccharification and fermentation, into one phase [85].

Using modified *A. terreus*, liquefied corn starch could be used more effectively for IA production compared to the *wild-type* strain [5]. At optimum conditions, using the most outstanding genetically engineered IA producer XH86-8, itaconate was produced at 77.6 g/L (after three days of fermentation) - which is approaching the industrial production yields [85,88].

The fast-developing microorganism *E. coli* is able to be a substitute for IA production with fewer by-products. In *E. coli*, with the recognition of common targets, a frequent problem can be solved, namely the chemical resistance of the microorganisms [95].

Several metabolically engineered *E. coli* strains have been reported recently [64,84,85]. The expression of α-amylase from *Streptococcus bovis* in *E. coli* produced after 69 h a quite low amount of 0.15 g/L itaconate using starch as substrate [85]. A production plasmid (pCadCS) that incorporates the codon-optimized (improved protein expression) *cadA* genes (*A. terreus*) and *gltA* (*Corynebacterium glutamicum*) were introduced in *E. coli* [84]. This strain produced 32 g/L itaconate and a maximum yield of 0.77 mol/(mol glucose). The whole cell bioconversion of citrate proposed by Kim et al. [96] can diminish the time and cost of itaconate production. Cys-aconitate decarboxylase (*cadA*) gene from *A. terreus* and *acn* from *C. glutamicum* with an improved expression at optimal conditions (pH 5.5 and 35 °C) produced 41.6 g/L itaconate. To achieve further increase in IA production using *E. coli*, glycerol was used as substrate, resulting in 43 g/L itaconate [97]. Compared to fungi, *E. coli* presents many advantages, specifically faster growth, easier handling, and accessible gene manipulation [85,97,98].

*A. niger* is an exceptional fungus in biorefinery usage since it can use a variety of substrates. *A. niger* is found to produce high quantities of citrate (180 g/L). Moreover, it is a promising strain in the production of itaconate as well. Through strain engineering and the application of a low-pH-induced-promoter that improves gene expression, *Pgas* in *A. niger* H915-1, 4.92 g/L IA was produced [88].

In *A. niger* with the overexpression of *cadA* or *mttA* genes from *A. terreus* and lowering the dissolved oxygen levels in the bioreactors, IA production increased from 0.8 g/L to 2.5 g/L [99]. With oxygen levels at 10%, the production of IA in *A. terreus* was 52.9 g/L, but with the expression of *cadA* gene from *A. terreus* in *A. niger*, the IA production was just 0.7 g/L [51]. The similar outcome obtained with the expression of the *cadA* gene in *A. niger* led to an increase from 0.05 g/L to 7.1 g/L IA production [42]. Therefore, the expression of the *cadA* gene in *A. niger* and through process optimization, IA production is achievable, but with the need for further studies to obtain higher yields [100].

### 2.5. Consolidated Bioprocessing

Compared to fossil oil, bio-catalytic conversion of cellulosic biomass is still more expensive for producing various compounds. A new process proposed to diminish these costs is consolidated bioprocessing (CBP)—which includes all three processes (cellulase production, hydrolysis of cellulose and fermentation of discharged sugars) in one step, or other pretreatment methods that apply fewer steps [101,102]. Antonov et al. [103] studied different cellulase producers and chose the most effective ones for IA production with *A. terreus*. Possible choices for cellulase production were *Trichoderma reesei* and *Penicillium verruculosum*. Although the observed faster cellulase production was with *T. reesei*; the overall results showed that *P. verruculosum* was a better choice when applying CBP, with higher carbon release rate and better cellulose digestibility.

Geiser et al. used the plant pathogen *U. maydis* MB215 strains [83] due to their special carbohydrate-active enzymes (CAZymes) that can degrade lignocellulose. These enzymes can be synthetically stimulated in the course of industrial development. In the production of itaconate, erythritol, hydroxyparaconate, and malate, different *U. maydis* strains have been used [104,105,106].

As future perspectives, investigation regarding new processes and strain developments to lower average costs while maintaining good yields of IA production is essential.

### 2.6. Recovery and Purification of IA

Improvements in the recovery and purification of IA are primary challenges in the advancement of biorefinery procedures [107]. The produced broth after IA fermentation contains residues, biomass, and other components, including organic acids [108]. The first step of IA recovery is the elimination of biomass and subsequently, the concentration and purification of IA. The primary separation and purification techniques used are precipitation, crystallization, adsorption, filtration, electrodialysis, and reactive extraction (Table 3).

A recent techno-economic analysis revealed that the achievement of the lowest production cost is with the adsorption method, but the drawback is the high amount of used water. Crystallization has a low yield of recovered IA, while requiring many steps and a high energy input. Electrodialysis and extraction need a lower energy input than crystallization but have higher costs. Extraction produces high amounts of wastewater, and electrodialysis consumes much energy [28].

As a conclusion, the technical feasibility of recovery and purification of IA is an essential challenge for achieving a high efficiency process.

## 3. Major Applications of IA

**Figure 3:**
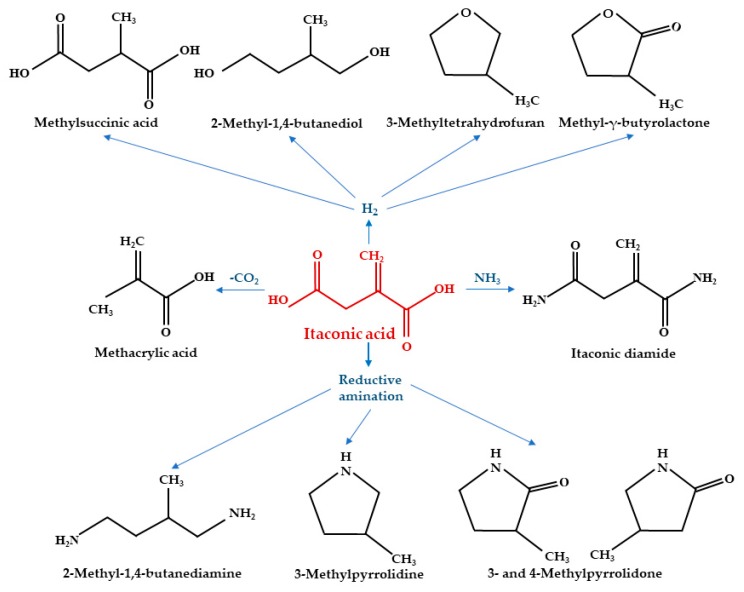
IA conversion into profitable derivatives.

Due to environmental problems with current plastics, producing degradable polymers and elastomers from bio-renewable resources is of significant interest [116,117]. The U.S. Department of Energy includes IA as one of the 12 building blocks that can be transformed into varied high-value bio-based chemicals or materials from biomass (Figure 3) [35,118,119]. Because of the development of biotechnological techniques and cost reductions (price <1 US$/kg), IA became a prospective commodity chemical [120].

Worldwide, IA primary usage is in styrene-butadiene rubber (SBR) latexes (44%), synthetic latex (9%), superabsorbent polymers (8%), chelant dispersant agents (7%), and in the production of methyl methacrylate (4%). Other promising uses are unsaturated polyester resins, phosphate-free detergents, and in the food industry [11,121]. IA is an ionic hydrophilic co-monomer that enhances the demeanor of antimicrobial release and adjusts the polymer arrangement [122]. IA presents a viable solution to replace acrylic acid in biodegradable polymers, and to act as a substrate for the production of methacrylic acid [17,123].

### 3.1. IA in Polymeric Hydrogels

**Figure 4:**
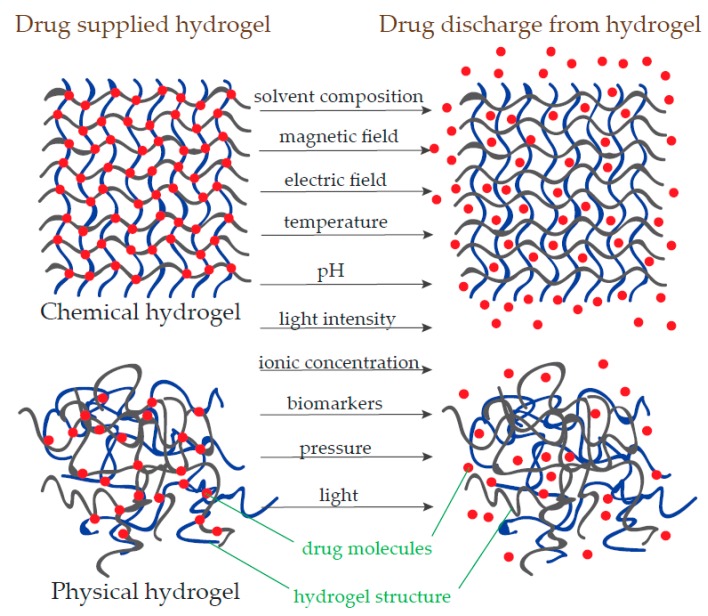
Hydrogel swelling and drug discharge with different physical or chemical stimulations.

Hydrogels have a structure composed of crosslinked polymer chains and can efficiently absorb considerable amounts of liquid when placed in aqueous solutions [124]. To qualify as a hydrogel, this hydrophilic polymer has to absorb at least 10% water [125]. These polymeric networks do not dissolve in water and are as flexible as the natural tissue. Hydrophilic gels exhibit specific traits of liquids like a high quantity of free liquid and solids with properly defined aspect. The soluble molecules can migrate throughout the gel. Due to the special properties of hydrogels, these polymers can be used in a wide diversity of products like super-absorbent polymers, tissue engineering, contact lenses, drug delivery, and biotechnological devices [126]. These polymers have the characteristics to increase, decrease, assign and discharge compounds when exposed to variations in particular environmental parameters such as temperature, ionic concentration, light intensity, pH, pressure or even at the effect of particular biomarkers [127,128]. Two distinct hydrogel categories are known, synthetic and natural hydrogels. By crosslinking type, hydrogels can be chemical and physical (Figure 4). Crosslinked structures with stable connections are chemical hydrogels and those resulted from polymer chain entanglements or other physical interactions are physical hydrogels [129,130]. Hydrogels are frequently used in both medical biology [131] and biotechnology [132] due to their capability to absorb high amounts of liquid.

Hydrogels made through polymerization as well as crosslinking of one or more polyfunctional monomers compose 3D networks. One possible natural source monomer is IA, together with its various esters, which can feasibly copolymerize with acrylamide and facilitate the swelling parameters of formed hydrogels. The highest swelling capability achieved with acrylamide and monomethoxyethyl itaconate hydrogel synthesized with a molar ratio of 70/30 was between 2,000 and 43,000% [127].

Amonpattaratkit et al. [133] studied high- and low-molecular-weight gelatin-methacrylate and gelatin-IA crosslinkers percentages for hydrogel microstructure, the improvement of biodegradation, crosslinking behavior, and swelling ratio characteristics of hydrogels. The most significant swelling ratio established was of 38 g/g with the 750 mM high-molecular weight gelatin-IA hydrogel. In comparison, the low-molecular-weight hydrogel had a slightly higher swelling ratio and faster degradation. With the increase of IA content, the swelling ratio increased in both hydrogels. As an outcome, the highest biodegradation characteristic, featured by a decrease of physical coherence and swelling medium integrity, occurred with the gelatin-IA hydrogel LG1750 of low molecular weight.

#### 3.1.1. IA Hydrogels Used in Water Decontamination

An essential application of biodegradable acrylamide hydrogels that include IA and itaconate groups in super absorbent hydrogels is in water-decontamination due to outstanding features such as low-price, precise handling, and reusability [124,134,135]. Water pollution presents a major worldwide problem that has caused serious environmental and health effects [136].

**Figure 5:**
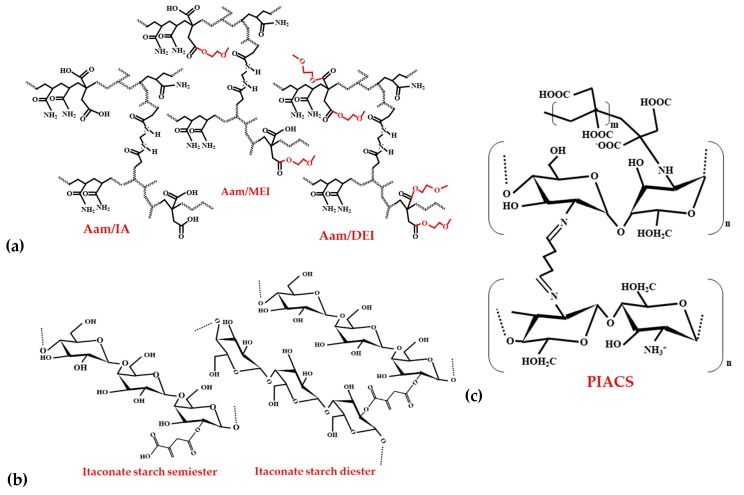
The chemical structure of IA synthesized hydrogels (**a**) Aam/IA, Aam/MEI, Aam/DEI (**b**) Itaconate starch semi- and diester (**c**) PIACS.

One of the major pollutants released in water comprises metallic ions like Fe^3+^, Pb^2+^, Cu^2+^, Cd^2+^, Fe^2+^, Cr^6+^, Ni^2+^, As^5+^, Cr^3+^, Zn^2+^, and Al^3+^ that arise from various industries and as an outcome of great industrial developments. Research comprising different itaconate hydrogel structures Aam/IA—poly(acrylamide-*co*-itaconic acid), Aam/MEI—poly(acrylamide-*co*-monomethoxyethyl itaconate), and Aam/DEI—(acrylamide-*co*-dimethoxyethyl itaconate) (Figure 5a) determined the effective adsorption of the following metal ions Cu^2+^, Fe^2+^, Fe^3+^, Pb^2+^, Ni^2+^ and Cd^2+^. Single metal ion adsorption was more efficient for the Aam/MEI hydrogel with Fe^3+^ than the hydrogel with Aam/IA. The adsorption capability in the multi-element sample was maximum with all the ions absorbed for both hydrogels at a molar ratio of 80/20. The trend of ion adsorption in both hydrogels was identical: Pb^2+^ > Cu^2+^ > Cd^2+^ > Ni^2+^ [132].

Starch is a naturally occurring biopolymer in high concentrations with several useful applications [137]. Soto et al. [138] generated starch-based superabsorbent materials with the natural compounds IA and maleic acid. The use of maleic acid presented high water solubility, and its use as an adsorbent was difficult. Itaconate starch mono- and diester presented competent crosslinking and substitution degrees. Itaconate starch diester hydrogel separated the metal ions Pb^2+^, Cd^2+^, Ni^2+^, and Zn^2+^ in the concentration of 11.24, 7.11, 5.10, and 8.44 mg/g and with a retention capacity of 145.03, 82.78, 56.74, 101.44 mg/g individually. Itaconate starch semiester hydrogel presented higher separation (25.16, 7.11, 5.10, 8.44 mg/g) and retention capabilities (506.44, 89.09, 72.99, 92.89 mg/g). Additionally, the same authors obtained novel hydrogels through graft copolymerization of starch with IA utilizing a redox system as the initiator (KMnO_4_/NaHSO_3_) [139]. This hydrogel could separate higher amounts of the same metal ions Pb^2+^, Cd^2+^, Ni^2+^, and Zn^2+^ with concentrations of 13.5, 13.0, 28.1, and 11.5 mg/g hydrogel, respectively. The retention capacities were 184.7, 175.0, 637.6, and 148.5 mg/g, respectively.

Other studies also proved that different IA hydrogels are useful in metallic ion adsorption [140]. Chitosan is another renewable biopolymer extensively studied for the preparation of natural hydrogels. With the new poly (IA)-grafted crosslinked chitosan nanomaterial—PIACS (Figure 5c) the adsorption capacity was 870.1 mg/g for Hg^2+^ and 1320 mg/g for Pb^2+^. The pH of the initial solution, contact time, dosage of adsorbent, and the initial ionic concentration had a strong influence on the high uptake of heavy metal ions. The regeneration of PIACS hydrogel loaded with heavy metals is possible with EDTA, after which it can be continuously used in adsorption-desorption phases [141]. The poly [2-(acrylamido)-2-methyl-1-propanesulfonic acid-*co*-IA] copolymer separated Pb^2+^, Cu^2+^, and Cd^2+^ ions in amounts of 1.74, 1.25 and 1.15 mmol/g polymer, respectively [142]. The inclusion of IA in the co-polymer had a positive effect on the adsorption of Cu^2+^.

The presence of Mn^2+^ in surface and groundwater is polluting for the environment and toxic to many living organisms, including humans. The nanomagnetite-charged poly (acrylamide-co-IA) hydrogel presented a competent and cheap Mn^2+^ elimination method from polluted waters [143]. The efficiency of Mn^2+^ removal in 60 min batch method was >99% at 25 °C and pH 6, and that presents strong results for additional research.

The hydrogels mentioned above had reusable characteristics without any measurable loss in the adsorptive capability throughout the adsorption-desorption cycle. In water or wastewater treatment, there is growing interest in the utilization of hydrogels that offer positive results in the removal of aqueous pollutants. The copolymerization of IA with different natural biopolymers usually improves the hydrogel structure and increases the degree of crosslinking [141,144]. Nevertheless, the essential challenges are in resource recovery, regeneration, reusability, and recovery of hydrogels [145].

#### 3.1.2. Bio-Based Smart Nanohydrogels for Food Applications

The creation of food packaging aims to preserve food from oxygen, microorganisms, light, and other environmental conditions. Petroleum-based synthetic polymers presently dominate the packaging industry. Active packaging acts positively on the extension of food preservation and enhances its quality. The primary usage of renewable IA in the food industry is in active packaging like smart nanohydrogels and in the delivery of food preservatives [122,146,147,148].

**Figure 6:**
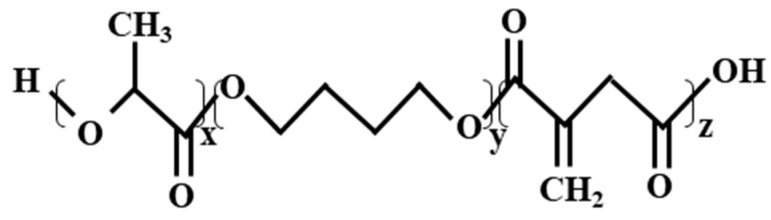
Poly(d,l-lactic acid-1,4-butanediol–itaconic acid) copolymer obtained from polycondensation of d,l-lactic acid, 1,4-butanediol, and IA.

Bio-based lactic acid and its copolymers also present a valuable alternative building block in the production of synthetic polymers. Through bulk polycondensation, a copolymer was synthesized from lactic acid, butanediol and IA, poly(d,l-lactic acid-1,4-butanediol-itaconic acid) (Figure 6). Through dispersion on a commercial folding boxboard’s backside, the obtained coating was perfectly grease-resistant and exceeded the commercial PLA coatings in performance. This PLA-based polymer (PLABDIA) can be used, especially in packaging dry and greasy goods [149].

The use of agricultural wastes as a substitute in the production of natural polymers encourages environmental sustainability. The high quantity of rice husk residues usage as immobilization carrier presents promising future applications. Functionalized rice husk was used recently as a new and inexpensive polymeric carrier for covalently immobilized enzymes (e.g., lipase B from *Candida antarctica*), and its effectiveness was compared to a classical enzyme carrier (an epoxy methacrylic resin). Lipase B is a broadly used enzyme in the cosmetic, polymer and food sector. When immobilized on rice husk, lipase B proved to be more efficient in solvent-free polycondensation of dimethylitaconate and 1,4-butandiol, than when it is immobilized in the epoxy methacrylic resin [150].

Molecularly imprinted hydrogels production with IA and 2-hydroxyethyl methacrylate present a positive result in biofilm inhibition. IA-based polymers reduced the *Pseudomonas aeruginosa* biofilm formation by a small amount compared with 2-hydroxyethyl methacrylate-based polymer [151]. Further studies in functional monomer concentration usage and optimizations are required for applying IA on equipment in food processing.

#### 3.1.3. Nanohydrogel Application in the Pharmaceutical Industry

Based on the World Health Organization (WHO) reports and according to a systematic analysis published in Lancet, infections are the second leading reason for global fatality [152,153]. This is mostly due to the antibiotic resistance of pathogenic microbes. In the antimicrobial field, hydrogels are useful biopolymers because of their high absorption capacity and delayed/controlled drug release [154]. According to the literature, IA and its derivatives are novel comonomers used in the preparation of a variety of pH-sensitive microgels in anti-tumor drug delivery [155,156,157]. IA is a remarkably hydrophilic antimicrobial agent, and it is capable of building hydrogen bonds with analogous groups. With the addition of different amounts of IA and crosslinking agents, drug release and drug-loading quantities can be efficiently controlled [131]. Also, due to pH responsiveness, this comonomer is efficiently used for drug delivery in the gastrointestinal tract [158].

The introduction of IA in polymeric chains improves the pH-sensitive and complexation character of the hydrogel. The enhanced swelling behavior owed to the two carboxylic groups in its structure reacts to electrostatic repulsion at appropriate pH and medium [159]. IA-based polymeric hydrogels accomplished the best results in inhibiting the *Candida albicans* fungus. This presents a promising application in vaginal infections, even without any antibiotic. The best swelling capacity, due to carboxylate anion construction obtained at pH 7.4-10, supports the usage in the biomedical field (neutral pH body fluid). This hydrogel is also used efficiently in wound treatment to protect against infections. Hydrogels polymerized with IA are nontoxic and 88% biodegradable under natural processes (when buried in soil for 90 days). With the increase of IA content in the hydrogels, the biodegradation promotion occurs due to an ester group in the IA hydrogel structure. A wide variety of microorganisms like fungi, bacteria, protozoa acted on the hydrogels under aerobic degradation. After degradation, H_2_O, CH_4_, CO_2_ and other compounds were produced [160].

A study with ampicillin and IA grafted tragacanth gum demonstrated that the therapeutic effect was better when ampicillin was loaded in the nanohydrogel. The release rate and the reaction against *E. coli* were more evident: with standard control the inhibition zone was 15 mm, and with the studied nanohydrogels the diameter was 19.3 mm, supporting the usage of these promising nanohydrogels in the administration of different antibiotics [161]. An in vitro study of paracetamol discharge, an exceedingly soluble and permeable drug, also disclosed interesting results through free radical copolymerization of IA and acrylamide. The increase of IA and reduction of the crosslinking agent positively influenced the water and drug uptake of the investigated hydrogels [131].

**Figure 7:**
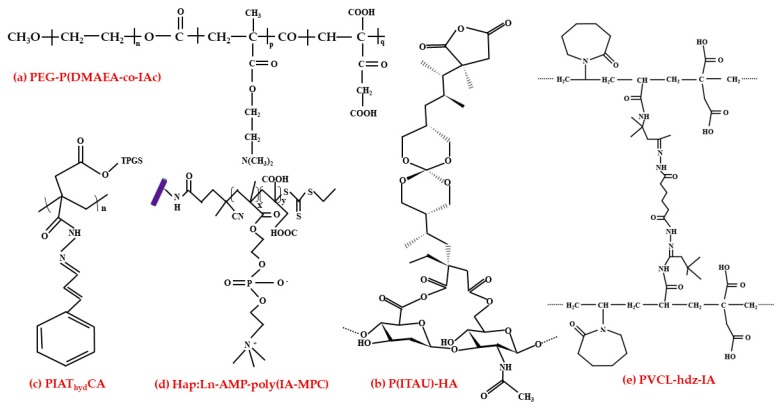
The chemical structure of IA synthesized nano-scale carriers (**a**) PEG-P(DMAEA-*co*-IAc), (**b**) P(ITAU)-HA, (**c**) PIAThydCA, (**d**) HAp:Ln-AMP-poly(IA-MPC), (**e**) PVCL-hdz-IA.

Cancer is one of the most widespread health burdens. Cancer cells have tenacious characteristics and can quickly adapt to unfavorable environmental conditions. Cancer is frequently treated with chemotherapy [162]. Due to the aggressive side effects and low cellular uptake of chemotherapeutic drugs in cancer cells, a possible solution for this problem is the usage of nano-scale carriers to enhance anticancer therapy. With a pH-responsive multi-drug nanocarrier, the administration of doxorubicin (an anticancer drug) and betanin (natural bioactive compound) were facilitated. Cancer cells have lower pH values than healthy cells; this way, the pH-responsive methoxy poly (ethylene glycol)-poly (2-dimethylamino) ethyl methacrylate-co-itaconic acid PEG-P(DMAEA-*co*-IAc) nanoparticles support regulated drug discharge at the pH values of cancer cells (Figure 7a). The use of this nanocarrier, with the simultaneous distribution of betanin and doxorubicin, showed improved cell uptake results, and also the cell apoptosis started [163]. Another bioactive chemo preventer multi-drug carrier system, namely poly (itaconic anhydride-*co*-3,9-divinyl-2,4,8,10-tetraoxaspiro (5.5) undecane, together with hyaluronic acid P(ITAU)-HA enhanced with quercetin (pro-apoptotic activity on tumor cell lines) and diclofenac (nonsteroidal anti-inflammatory drug) proved to be more reactive to environmental stimuli (Figure 7b). The investigation through in vivo studies of six types of hydrogels with different concentrations of P(ITAU) revealed the best results with 20% of this matrix. In addition, the swelling degree reliance of the studied “intelligent” gel structures on temperature, pH and environmental stimuli demonstrated significant features [164].

A recent study constructed a system with free radical polymerization of IA to poly (IA) D-α-tocopherol polyethylene glycol 1000 succinate and cinnamaldehyde (PIAT_hyd_CA) through a hydrazone bond (_hyd_) (Figure 7c).The aim of this study was to increase the intracellular reactive oxygen species level that can eliminate tumor cells (activate apoptosis). Regarding this system, the cinnamaldehyde (sensitive to pH) became more bioavailable and, together with the D-α-tocopheryl polyethylene glycol 1000 succinate, activated tumor cell apoptosis. In anticancer treatment, this method is most effectively used due to the mitochondrial apoptosis pathway against MCF-7 breast cancer cells [165]. Owing to their bone-like chemical composition, rare-earth ions doped hydroxyapatite nanoparticles are very efficient in biological imaging. The nanoparticles with adenosine 5′-monophosphate disodium salt and IA polymerized with 2-methacryloyloxyethyl phosphorylcholine—HAp:Ln-AMP-poly(IA-MPC) (Figure 7d) have a usage in cancer treatment by loading the anticancer drug cisplatin. These polymeric composites had unique properties with adequate diffusion in liquids, functional pH dependency, efficient drug distribution, and adverse toxic effect against A549cells. The composites mentioned above can get through the cell wall and help in drug delivery and the dispersion of active components intracellularly [166]. A two-fold sensitive biodegradable microgel synthesized through precipitation polymerization, namely poly(*N*-vinyl-caprolactam-*co*-itaconic acid)—PVCL-hdz-IA (hydrazone-based crosslinking) (Figure 7e), is responsive to temperature and pH. PVCL-hdz-IA supplied with doxorubicin discharged the drug favorably at acidic circumstances. With the growth of IA quantity, the volume-phase transition temperature increased. The storing capacity of PVCL-hdz-IA microgel reached 14.1%, acquired through the hydrogen bond interaction and electrostatic energy of doxorubicin and polymer and with exceptional stability at pH 7.4. The annihilation of human cervical carcinoma cells (HeLa cells) was very powerful with doxorubicin-encapsulated microgel, with a viability of approximately 90%; without the encapsulated drug only 22.6% were viable [167].

Through the administration of eye-drops, the bioavailability of drugs is very low; to overcome this problem, the production of soft contact lenses may be a good alternative. Eroglu et al. [168] developed minocycline-imprinted hydrogels with distinct functional monomers to treat optical disorders like glaucoma, dry eye, and conjunctivitis. These functional monomers were acrylic acid, methacrylic acid, IA, 1N ethylacryl amide, 1N isopropyl acrylamide, and hydroxymethyl acrylamide. From the used functional monomers regarding hydrogen bonding with IA, the acquired results were meaningful and indicated their high potential as minocycline imprinted hydrogels. Nevertheless, acrylic acid is a more common monomer used in contact lenses manufacturing; with IA, no further studies were performed. Dosimetry methods need to be accurate and reliable, as it contributes to treatment verification in ionizing radiation. A recent study for radiology usage analyzed the water-equivalence properties of Fricke (cheaper and easier gel dosimeter method) and three different polymer gel dosimeters. Each of the polymer applications in gel dosimeters like IA, N-isopropyl acrylamide, and polyacrylamide presented the same linear trend at a dose-response curve, as well as a raised intensity in water-equivalence [169].

### 3.2. Antimicrobial Agent and Medical Applications

Antibiotic-resistant bacteria causes many bacterial contaminations, which claim a considerable number of lives every year in Europe and the USA (~50,000 people) [170]. Antimicrobial peptides found in almost every organism`s immune system help to react rapidly to pathogens. Based on nature, nowadays there is in progress an ongoing study on synthetic antimicrobial peptides. These polymer coatings need to be long-term stable and easily secured on medical devices [171]. Asymmetrically disubstituted diitaconate monomers copolymerized with dimethyl acrylamide propose an encouraging material against bacterial infections as synthetic mimics of these antimicrobial peptides. The easily polymerized IA is a harmless and ecological source that can also prevent bacterial contamination [172]. The polymers mentioned above are relatively cheap (synthesized by metal-free initiator systems), but further structural optimization is still in progress.

The use of IA salt through the introduction in beverage or food acts as a glycolytic pathway metabolic regulator with antidiabetic, antiobesity, and/or antilipemic consequences. Administration of IA in drinking water to rats revealed diminished weight gain, free fatty acid, blood glucose, and triglyceride levels. These results proved the effectiveness against hyperlipidemia and diabetes in rats. Although IA, along with its salts, was classified as harm-free in these animals with no negative effects, it is still not used in drinks, foods, or medical products [173].

Gestational diabetes mellitus occurs during pregnancy and presents unfavorable severe health effects for mother and child. The screening of disease susceptibility accomplished at the beginning of pregnancy with the detection of biomarkers and some possible metabolic mechanisms demonstrate successful prediction tools. IA was proposed as a novel biomarker because of its presence at a higher concentration in the serum of women at 20 weeks’ pregnancy who afterward developed gestational diabetes mellitus compared to controls with uncomplicated pregnancies, which did not develop diabetes [174]. Meiser et al. [175] found that the detection of IA in patients infected with sepsis was impracticable. The analysis of urine and blood samples failed to identify IA at an acceptable concentration in the circulation. As a consequence, this study demonstrated that IA currently has no potential as a biomarker in body fluids (not actively excreted), but its use in immune cells as a pro-inflammatory marker is possible (intracellularly abundant) [175].

A study demonstrated that itaconate abolishes visceral fat from glucose in rats through the reduction of fructose 2,6-bisphosphate, which aftermath leads to the restriction of liver glycolysis. IA in rats, due to this phosphofructokinase restriction, may also be associated with metabolism adjustment. IA metabolization rate is slow, which maintains its impact on glycolysis through the lessening of 2,6-bisphosphate quantity [176]. Recent data present that itaconate has a bacteriostatic outcome in macrophage mobilization, such as *Mycobacterium tuberculosis* or *Salmonella enterica.* IA is synthesized by the enzyme encoded in the *Irg1* gene and is responsible for the macrophage antimicrobial activity [52] and anti-inflammatory effect through Nrf2 activation in mouse and human macrophages [177].

### 3.3. Other Applications

Due to the high concentration of styrene in commercial unsaturated polyester resins, these materials are suspected carcinogens, respiratory tract irritants, and neurotoxins, which can cause severe and prolonged effects on health [178]. To reduce the amount of styrene, bio-based unsaturated polyester resins are synthetized [179].

Nowadays, the high amount of plastic waste is a growing concern. The rise of plastic manufacturing is an ever-increasing global problem, which in 2015 reached approximately 322 million tones and with a quantity twice as large anticipated by 2035. For the reduction of the vast amount of these wastes, the European Commission proposed a strategy for plastics in a circular economy which also included the development of innovative and recycled plastics manufacturing [180].

In this regard, through crosslinking of bio-based unsaturated polyester resins (UPR), and functionalized PET particles (resulted by mechanical size reduction of recycled PET fibers obtained by extrusion) with the addition of a reactive diluent, namely dimethyl itaconate, a new composite material formed. The use of dimethyl itaconate had the purpose of improving the compatibility and interaction between the PET particles and UPR. Petrochemical-based UPRs have better mechanical properties than bio-based UPRs; on the other hand, due to their sustainability, low price, and low carbon footprint, these composites present desirable properties [181]. A research study with diluted dialkyl itaconate involving IA and 1,2-propanediol through melt polycondensation, synthesized effectively 100% green unsaturated polyester resins [179]. Nevertheless, the growth of the quantity of the itaconate ester group affected the mechanical properties of the polyester resins negatively. Actual bio-based unsaturated polyester resins have the decisive feature of not releasing volatile petrochemical and toxic elements. IA and its derivatives proved to be a low-cost and relatively easy method for the synthesis of these polymers.

## 4. Conclusions and Future Perspectives

Annually, the production of biomass wastes is in the millions of tons range. A possible solution for remediation of these environmental issues can be the biomass utilization in fermentations. Significant potential compounds obtained from bio-wastes are organic acids such as Itaconic Acid (IA), which can also be a substitute for non-renewable petrochemicals, like acrylic acid.

Many types of fungi like *A. terreus* and *U. maydis* can produce IA. Some bacteria like the genetically engineered *E. coli* and *C. glutamicum* can also produce significant quantities of IA. IA presents many advantages over fungi, specifically faster growth, easier handling, and accessible gene manipulation. Further research regarding inhibitory components, metabolic regulation and operation conditions to increase the IA yield, is necessary. IA has many novel applications owing to its unique characteristics, namely the structural resemblance to acrylic acid, methacrylic acid and due to an α, β-unsaturated functionality that allows for the formation of new polyesters.

Overall, the present manuscript attempts to present the diversity of novel applications of IA as biodegradable hydrogels in water decontamination with unique features efficient in metallic ion adsorption and in smart packaging that can hold food preservatives. In the medical industry, the primary utilization of IA is in controlled drug delivery, especially as multi-drug nanocarrier with great effect on apoptosis of tumor cells. Other medical usages are in wound treatment, optical disorders, in radiology for gel dosimeters and many other applications. Bio-derived polyesters from IA mainly used in elastomer production as a cross-linkable constituent can replace styrene, which has adverse health effects. IA also has an antimicrobial effect.

Lignocellulosic wastes are promising and renewable substrates for IA production, but there is a demand for more studies to make it profitable as an alternative for the traditional methods. Ecological IA-based polymers have abundant application possibilities, and further studies to find other innovative polymers are still required.

## Figures and Tables

**Table 1:** Fermentation parameters and yields using different *A. terreus* strains.

Substrate	*A. terreus*	IA [g/L]	pH	T [°C]	Method	OP [g/L/h]	Y [g/g_TS_]	Ref.
glucose	NRRL 1960	24.7–49.5	1.8–2.0	34	BF	0.33–0.44	N.A.	[22]
carbon sources	NRRL 1960	129	2.1–6.0	N.A.	SF	N.A.	N.A.	[19]
glucose			2.5	35	BR	N.A.	N.A.	[71]
corn starch	TN-484	62	1.5	30	SF/ALB	N.A.	N.A.	[72]
		61	2.0	30				
		59	2.5	30				
		57	3.0	30				
Jathropa seed cake		24.46	3.5	32	SF	N.A.	N.A.	[73]
glucose		86.2	3.1	33	STR	0.51	0.62	[66]
		90				N.A.	0.58	
corn starch	CICC 40205	77.6	4.0	37	SF	N.A.	N.A.	[62]
glucose	DSM 23081	129	3.1	33	STR	N.A.	N.A.	[68]
		87	N.A.	N.A.				
		146	3.0	N.A.				
rice husk	ATCC 10020	1.9	6.0	30	SF	N.A.	N.A.	[63]
wheat chaff	DSM 23081	27.7	3.1	33	SF	0.19	0.41	[45]
artif. wheat chaff		51.5	3.1	33		0.31	0.59	
potato starch	C1	30.8	N.A.	35	SF	N.A.	N.A.	[61]
	C2	23.4	N.A.	N.A.				
wheat bran	CICC 40205	49.65	7.0	32	SF	N.A.	N.A.	[74]
mannose	NRRL 1971	36.4	3.1	33	SF	N.A.	0.46	[30]
glucose		42.6					N.A.	
xylose		30.5						
arabinose		25.8						
galactose	DSM 23081	9.1						
glucose	DSM 23081	129	3.0	35	SGR	0.61	0.57	[75]
		138	3.2			0.82	N.A.	
		162	3.4			0.99	0.46	
		150	3.0		STR	N.A.	0.56	
glucose	DSM 23081	70	3.1	33	Fl	N.A.	N.A.	[76]
glucose	DSM 23081	105	3.1	33				
glucose	NRRL 1960	51.9	3.1	33	SF	N.A.	N.A.	[77]
xylose	DSM 23081	38.7						
arabinose	NRRL 1961	34.8						
GXA		33.2						
glucose	NRRL 1960	73.6	3.0	33	BF	N.A.	0.85	[44]
fruit waste	SKR10	20	3.0	34	SF	N.A.	0.22	[78]
Corn starch		28.5–31.0				N.A.	0.26	

OP—Overall productivity, Y—Yield, TS—total sugar, SF—shake flask, F—fermenter, BF—biofermenter, Fl—Flask, STR—tank reactor, BR—batch reactor, SGR—stirred glass reactor, ALB—air-lift bioreactor, N.A.—not available.

**Table 2:** Fermentation parameters and yields using different methods and microorganisms.

Substrate	Strain	IA [g/L]	pH	T [°C]	Method	OP [g/L/h]	Y [g/gTS]	Ref.
glucose	*Yarrowia lipolytica*	4.6	3.5– 5.0	28	BR	0.045	0.058	[49]
glucose	Candida sp.	30-35	3	26	flask	N.A.	N.A.	[80]
glucose	*C. glutamicum* strains	1.4–3.6	7.0	30	BSF	N.A.	0.011	[81]
urea		13.3–59.0					0.23	
glycerol	*U. vetiveriae* TZ1	34.7	6.5	N.A.	SF	0.09	N.A.	[82]
glucose	*U. maydis* MB 215	4	6	30	CF	0.8	N.A.	[46]
potato starch	*U. maydis*	34.52	N.A.	N.A.	N.A.	N.A.	N.A.	[61]
glucose	*U. maydis* MB215	20	N.A.	30	SF	0.27	0.17	[79]
cellobiose	*U. maydis* strains	N.A.	6.5	30	SF	N.A.	N.A.	[83]
glycerol	Eng. *E. coli*	22	5–5.5	37	BSF	0.6	0.55	[74]
xylose		20				0.6	0.51	
glucose		18				0.43	0.36	
glucose	*E. coli* strains	2.27	N.A.	37	SF	N.A.	0.77	[84]
		32	N.A.	30	BR	N.A.	0.68	
starch	*E. coli*	0.15–0.62	6.8	28	JF	N.A.	N.A.	[85]
glucose	*A. niger* strains	26.2	3.5	33	BF	0.35	N.A.	[43]
glucose	*A. niger* strains	0.26–0.29	3.5	33	BF	N.A.	N.A.	[86]
sorbitol	*A. niger* CAD4	3–8	N.A.	30	flask	N.A.	N.A.	[87]
sorbitol + xylose	Eng. *A. niger* + *cadA*	54.3	N.A.	30	F	N.A.	N.A.	[42]
glucose	Eng. *A. niger* strains	0.82–4.92	3.1	35	SF	N.A.	N.A.	[88]

OP—Overall productivity, Y—Yield, TS—total sugar, SF—shake flask, F—fermenter, CR—continuous fermenter, JF—jar fermenter, BF—benchtop fermenter, Br - bioreactor, Fl—flask, BSF—baffled shake flask, N.A.—not available.

**Table 3:** IA recovery method examples and main process disadvantages

Method	Media	Yield [%]	Disadvantage	Ref.
Crystallization	FB	80	- high thermal energy input required - reduced final purity > additional purification step	[37]
Crystallization	FB	51	- low yield - high thermal energy input required	[109]
Crystallization	FB	23	- low yield - change in fermentation temperature	[110]
Adsorption	AS	100	- high waste-water quantity	[107]
Reactive extraction/back extraction/pH- shift crystallization	FB	99	- NaCl salt by-product formation	[111]
Reactive extraction	AS	94.7	- decreased toxicity due to usage of vegetable oil	[112]
Back extraction		80	- high energy demand - unwanted side products	[113]
Reactive extraction	FB	91	- mass transfer area limitations	[76]
Reactive extraction	AS	80	- process under study	[114]
Electrodialysis	AS	50	- low efficiency - competitive only with a yield of 98%	[115]

FB—Fermentation broth; AS—Aqueous solution.
